# Mentorship: A key mission of the Women’s Dermatologic Society

**DOI:** 10.1016/j.ijwd.2015.04.004

**Published:** 2015-05-26

**Authors:** Dedee F. Murrell, William D. James

**Affiliations:** Department of Dermatology, St George Hospital, Sydney, Australia; Department of Dermatology, University of Pennsylvania

This issue’s “A piece of my mind” article by Dr. Fiona Cornish, immediate past president of the Medical Women’s Federation of the United Kingdom, speaks about the Women’s Dermatologic Society’s (WDS’s) “buddy” system of unofficial mentorship. It was this particular aspect of the WDS that drew me (DFM) into the WDS as a first-year dermatology resident, introduced by longstanding WDS member, Dr. Elise Olsen.

Within the WDS there are men and women who seem to love the mission of helping and guiding others, even if those people are not working with them. Dr. Frances Storrs was such a well-known mentor for contact dermatitis that when she was given the Rose Hirschler Award in 1991, she announced at the luncheon that she was donating her award money back to establish the first WDS mentorship grants. Today, the WDS gives out about 30 mentorship grants each year; the list of those who have received previous mentorship grants can be found at http://www.womensderm.org/?page=PastMentorshipRecpts.

These WDS Mentorship grants enable residents, fellows, and junior faculty of both sexes to spend a few weeks with a WDS mentor to up-skill in their areas of expertise. So long as either the mentee or mentor is female, this is suitable for consideration. The inclusion of men in this program provides a formal means for male mentors to support women’s careers, while male mentees gain insight into the viewpoints of successful women in medicine. WDS fundraisers have found this mentorship grant to be the most popular area of funding support to the WDS.

In 2003, the WDS began to recognise a “Mentor of the Year,” nominated by mentees. A list of these is given below:

2015 - Lisa A. Garner MD
  2014 - Mary P. Lupo, MD
2013 - Dedee F. Murrell, MD
2012 - William D. James, MD
2011 - Pearl E. Grimes, MD
2010 - Mary Maloney, MD
2009 - Tina S. Alster, MD
2008 - Jean Bolognia, MD
2007 - Libby Edwards, MD
2006 - Amy Paller, MD
2005 - Ilona J. Frieden, MD
2004 - Vera H. Price, MD
2003 - Frances J Storrs, MD

WDS believes in informal mentorship (i.e., not officially assigning new members to particular mentors). The networking reception is a great venue to meet new people in the WDS, as is the luncheon at the American Academy of Dermatology meeting. By generously including men in these venues, the WDS allows men interested in tackling gender inequity an informal opportunity to steward women through challenges and assist their advancement. Much of the mentorship consists of pearls of wisdom about how to manage your career with a husband in a busy career, how to juggle having children with your career, how to manage your office staff, etc. A few interesting ones that come to mind are: Wilma Bergfeld – always dress as you wish to be perceived; Joy Rico – despite your career, your children are the greatest gift you will ever receive; Susan Weinkle – have recipe cards so your home help can prepare dinner for you, don’t try to be superwoman.

